# Author Correction: Covalent penicillin-protein conjugates elicit anti-drug antibodies that are clonally and functionally restricted

**DOI:** 10.1038/s41467-024-52286-6

**Published:** 2024-09-11

**Authors:** Lachlan P. Deimel, Lucile Moynié, Guoxuan Sun, Viliyana Lewis, Abigail Turner, Charles J. Buchanan, Sean A. Burnap, Mikhail Kutuzov, Carolin M. Kobras, Yana Demyanenko, Shabaz Mohammed, Mathew Stracy, Weston B. Struwe, Andrew J. Baldwin, James Naismith, Benjamin G. Davis, Quentin J. Sattentau

**Affiliations:** 1 Sir William Dunn School of Pathology, University of Oxford, Oxford, OX1 3RE UK; 2 Rosalind Franklin Institute, Harwell Science and Innovation Campus, Oxford, OX11 0FA UK; 3 Department of Chemistry, University of Oxford, Oxford, OX1 3TA UK; 4 Kavli Institute for Nanoscience Discovery, Dorothy Crowfoot Hodgkin Building, University of Oxford, Oxford, OX1 3QU UK; 5 Department of Biochemistry, University of Oxford, Oxford, OX1 3QU UK; 6 Department of Pharmacology, University of Oxford, Oxford, OX1 3QT UK; 7 The Max Delbrück Centre for Molecular Medicine, Campus Berlin-Buch, 13125 Berlin, Germany; 8 Experimental and Clinical Research Center (ECRC), Charité Universitätsmedizin Berlin and Max-Delbrück-Center for Molecular Medicine, Lindenberger Weg 80, 13125 Berlin, Germany; 9 Present Address: Laboratory of Molecular Immunology, The Rockefeller University, New York, NY 10065 USA

**Keywords:** Immunology, Chemical biology, Antibodies, Structural biology, NMR spectroscopy

Correction to: *Nature Communications* 10.1038/s41467-024-51138-7, published online 10 August 2024

The original version of this Article contained an error in the spelling of the author Yana Demyanenko, which was incorrectly given as Yana Demyaneko. In addition, the original version of this Article contained errors the main text as follows:

In the incorrect statement ‘PenG has constituent β-lactam, thiazolidine and benzylamide side-chain moieties (Fig. 1a);’ The correct version states ‘phenylacetamide’ in place of ‘benzylamide’.

In the incorrect statement ‘Our analysis focuses on the H/L molecules (Fig. 5f). The PenG benzene group is deeply buried’, ‘benzene‘ should be replaced by ‘phenyl’.

In the incorrect statement ‘The interaction with Tyr68L has strong π-stacking character whilst the planes of the rings benzene and Tyr110L’, ‘rings benzene’ should be replaced with ‘rings found in the phenyl moiety’.

In the figure legend of Fig. 5, ‘phenylacetamide’ should replace ‘phylacetamide’ in the incorrect statement ‘Key residues within 4.0 Å of ligand aspects ii. phylacetamide’.

The original version of this Article contained an error in Fig. 5a, in which there was an error in the displayed bonds of a chemical structure. The correct version of Fig. 5a is:
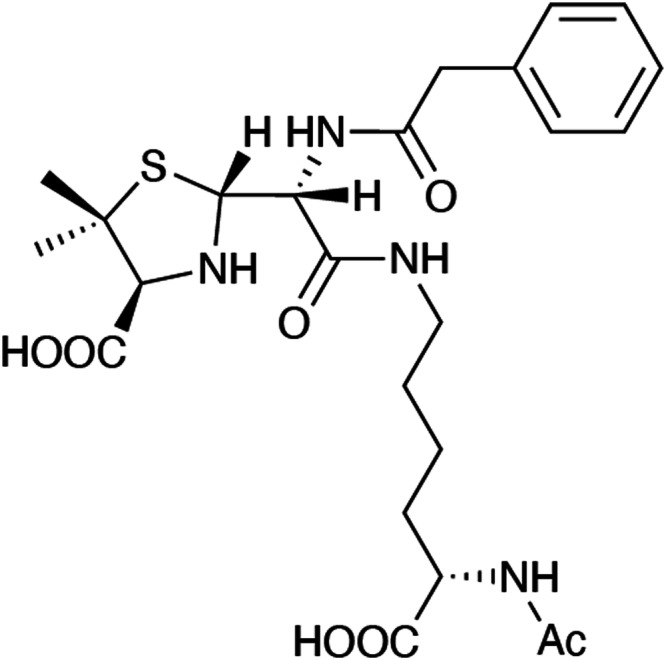


which should replace the previous incorrect version:
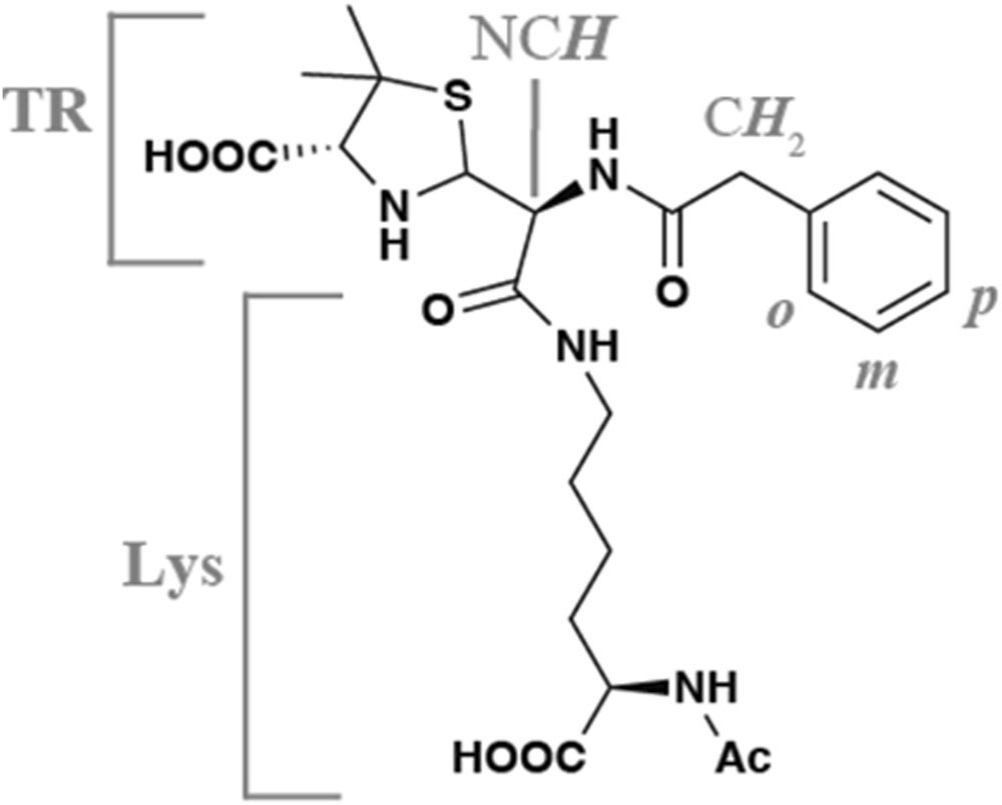


These have been corrected in the HTML and PDF version of the Article.

