# Supplementary material for: Covalent penicillin-protein conjugates elicit anti-drug antibodies that are clonally and functionally restricted
Source: Nat Commun. 2024 Aug 10;15:6851. doi: 10.1038/s41467-024-51138-7 (PMC11316840; doi:10.1038/s41467-024-51138-7)
Supplement: Supplementary file 1 — Supplementary Information [file 41467_2024_51138_MOESM1_ESM.pdf]

Figure S1: Chemical characterisation of HEL-PenG and its immunogenicity in mice.

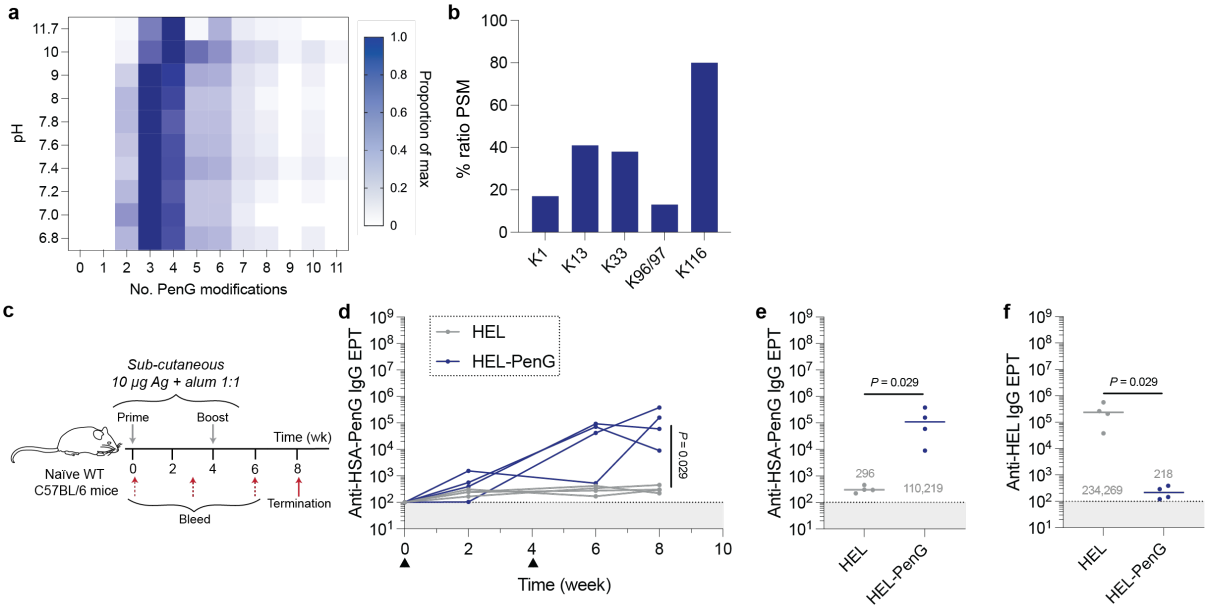

(a) The PenG adduct occupancy was tested with respect to buffer condition, evaluating the relative number of adducts via mass spectrometry. **(b)** Site-specific occupancy of PenG adducts on HEL-PenG (modified at pH = 8.0). **(c)** Immunisation schedule. Sex-matched WT 6-week-old C57BL/6 mice were immunised with 10 µg/mL HEL or HEL-PenG in alum. IgG antibody titres against the PenG adduct were evaluated by screening cross-reactivity against HSA-PenG. Created with BioRender.com released under a Creative Commons Attribution-NonCommercial-NoDerivs 4.0 International license. This was conducted both **(d)** longitudinally and **(e)** at the terminal timepoint. **(f)** The terminal protein backbone-specific response was also determined. Dots represent values from a single animal (*n* = 4) and bars denote the median. Groups were compared via Mann-Whitney tests.

Figure S2: PenG occupancy on HSA.

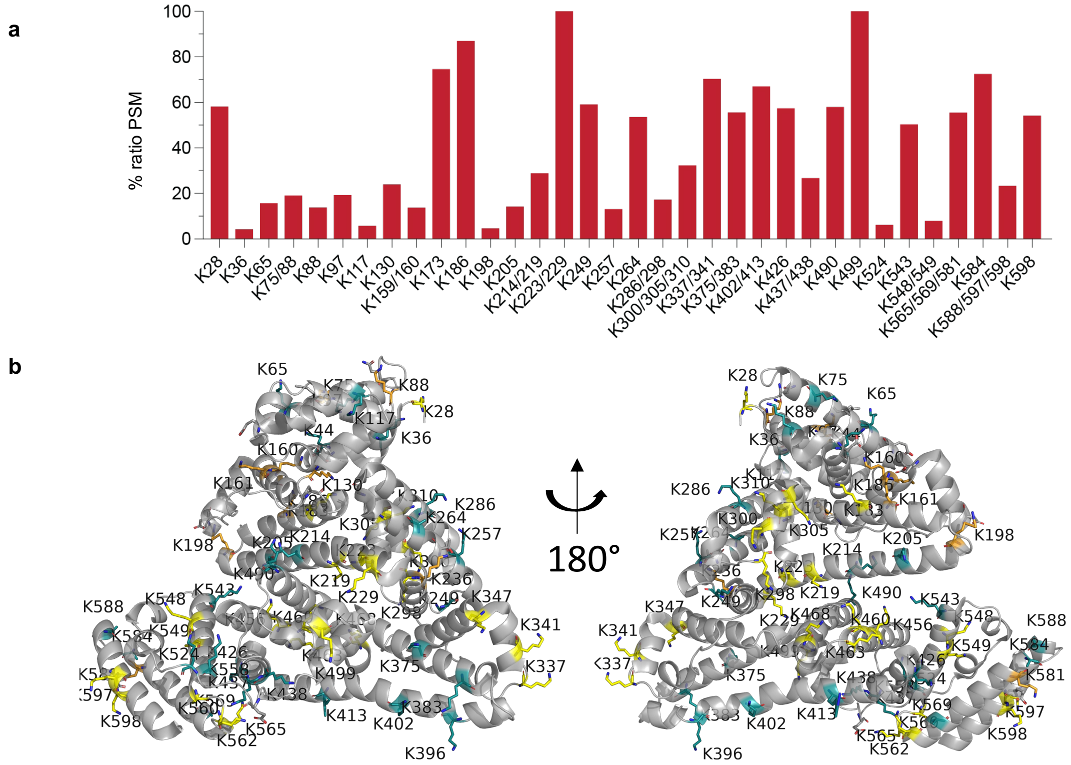

Site-by-site occupancy of PenG adducts on HSA-PenG (modified in 0.1 M Na_2CO_3, pH = 8.0) determined by tryptic digestion and subsequent evaluation via LC-MS/MS. **(a)** Approximation of Lys modification levels by site using the ratio of peptide spectrum match for PenG-reacted and unreacted samples. It should be noted that variation of PSM can also arise from modulated peptide occurrence arising from restricted digestion or overdigestion to unobservable fragments. **(b)** Representation of MSMS analysis of Lys reactivity coverage in HSA on the structure of HSA [pdb 6hsc]. Coloured lysines represent mapped reactivity using the proxy of reduced peptide ion intensity of unmodified peptide in PenG treated sample compared to control. Teal with stick representation of side chain: those with reduced intensity of the unmodified perfectly cut peptide compared to control, consistent with modification by PenG. Orange with stick representation of side chain: those where the intensity of the original peptide was not appreciably reduced compared to control. Yellow: those that are unclear due to local sequence (e.g. unmodified peptide on either side is too short to be identified in control). Interacting residues (mostly Asp and Glu, with some backbone interactions) are also shown as an indication of accessibility. Modifed lysines are generally more solvent exposed and their ε-N are not immediately involved in intramolecular interactions, whereas unmodified lysines are mostly buried and have less accessibility or are engaged by Asp or Glu.

Figure S3: Memory splenocyte responses against PenG-adduct-immunized mice.

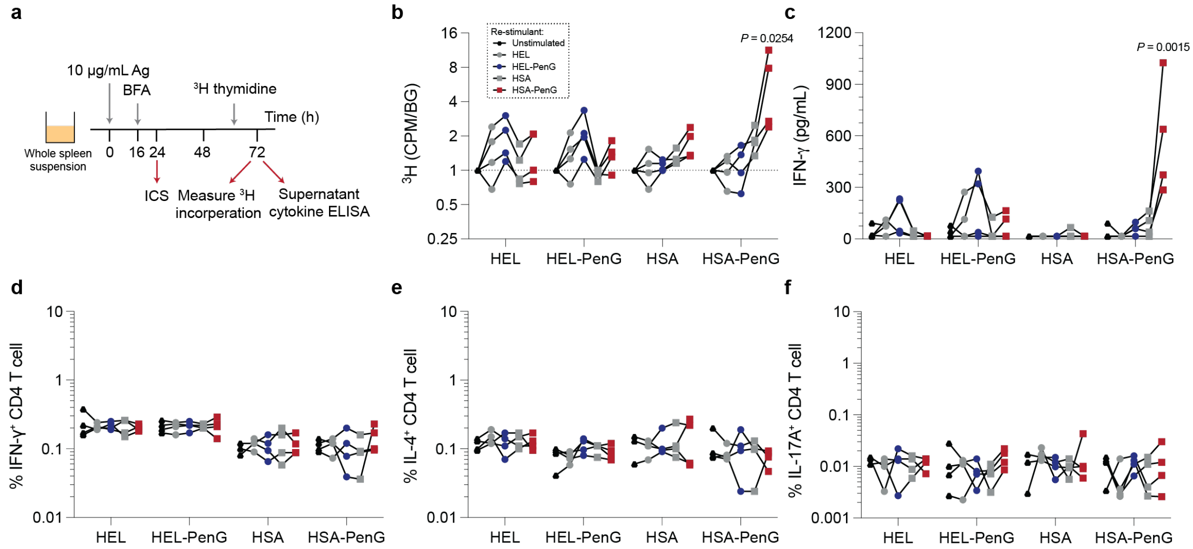

These experiments were conducted on spleens harvested from mice twice immunized with antigen in alum (weeks 0,4) and spleens harvested following 8 weeks. **(a)** Experimental pipeline for *in vitro* splenocyte re-stimulation assays**.** Created with BioRender.com released under a Creative Commons Attribution-NonCommercial-NoDerivs 4.0 International license. **(b)** Incorporation of 3H-thymidine was measured and normalised to background. Relative counts per million were presented on a $\log_2$ scale**. (c)** IFN-γ production in cell supernatant was measured via ELISA. ICS was conducted on re-stimulated splenocytes, reporting the percentage of **(d)** IFN- γ^+, **(e)** IL-4$^+$ and **(f)** IL-17A$^+$ CD4 T cells. Data were compared via paired tests (n = 4).

Figure S4: PenG occupancy of MSA.

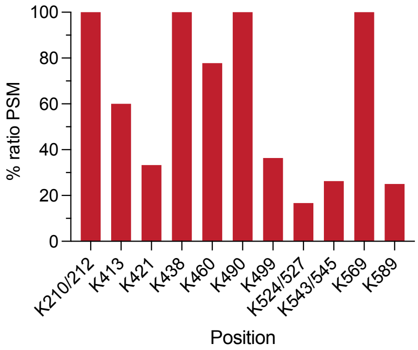

Site-by-site occupancy of PenG adducts on MSA-PenG (modified in 0.1 M Na_2CO_3, pH = 8.0) determined by tryptic digestion and subsequent evaluation via LC-MS/MS. Approximation of Lys modification levels by site using the ratio of peptide spectrum match for PenG-reacted and unreacted samples. It should be noted that variation of PSM can also arise from modulated peptide occurrence arising from restricted digestion or overdigestion to unobservable fragments.

Figure S5: Serological responses following the administration of free PenG/PenV.

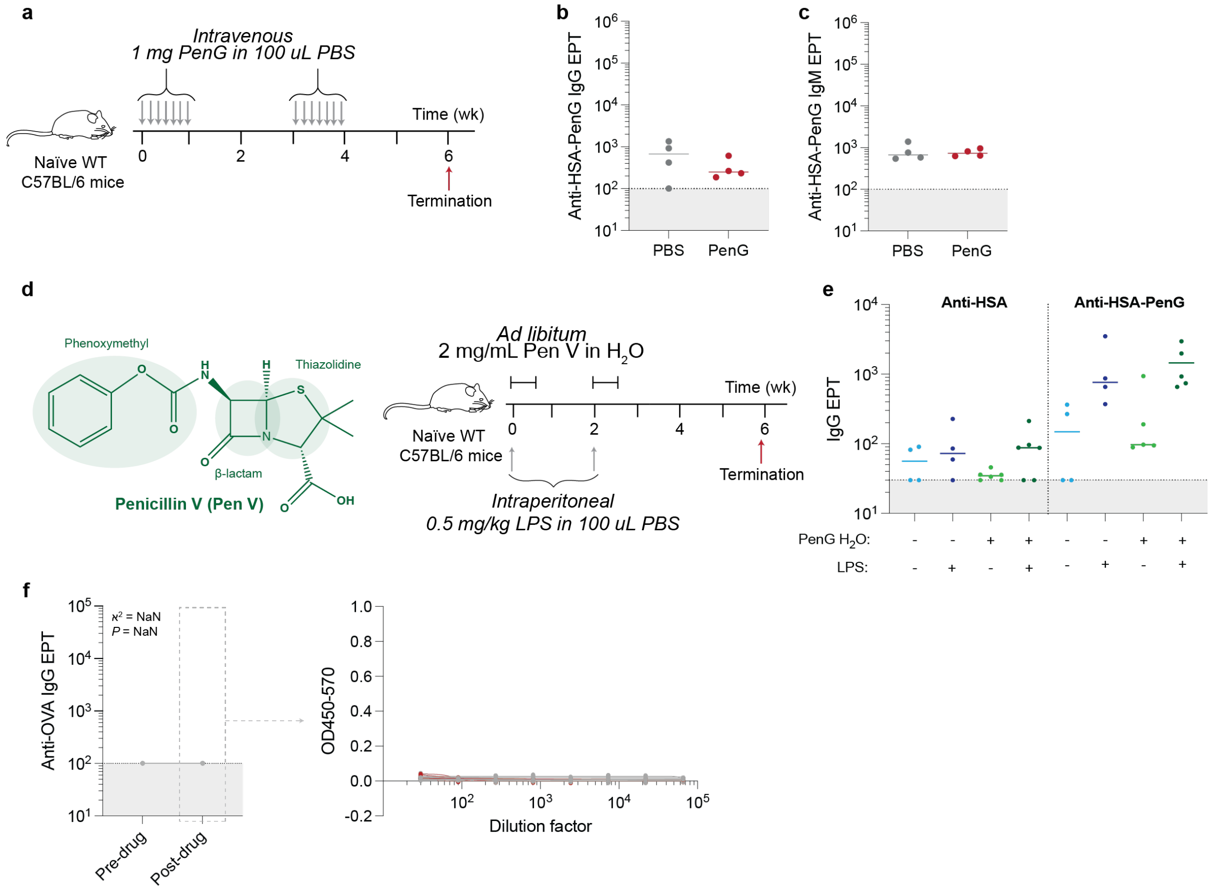

(a) Mice received two week-long courses of PenG intravenously. **(b)** Terminal IgG and **(c)** IgM EPTs were determined against HSA-PenG. **(d)** PenV in water at 2 mg/mL was given to animals *ad libidum*. Some mice revied intraperitoneal immunisations of LPS. **(e)** IgG EPTs against HSA and HSA-PenG were evaluated. **(f)** Anti-OVA EPTs of mice given PenG-Ben intramuscularly. Dots represent data from a single mouse (n = 4–6; 27), and bars denote the median. **(a, d right)** Created with BioRender.com released under a Creative Commons Attribution-NonCommercial-NoDerivs 4.0 International license.

Figure S6: Free-amines following β-lactam modification.

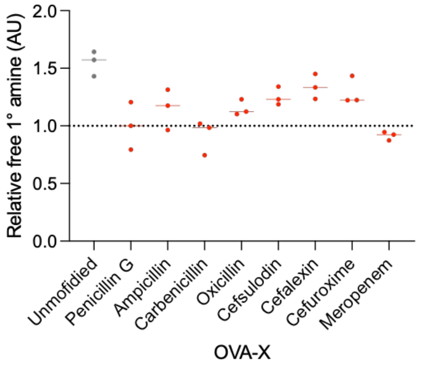

Relative free primary amine was quantified on OVA using a 2,4,6-trinitrobenzenesulfonic acid-based method, following alternative β-lactam modification. Data acquired using three technical replicates.

Figure S7: V_H gene segments in PenG-specific B cells.

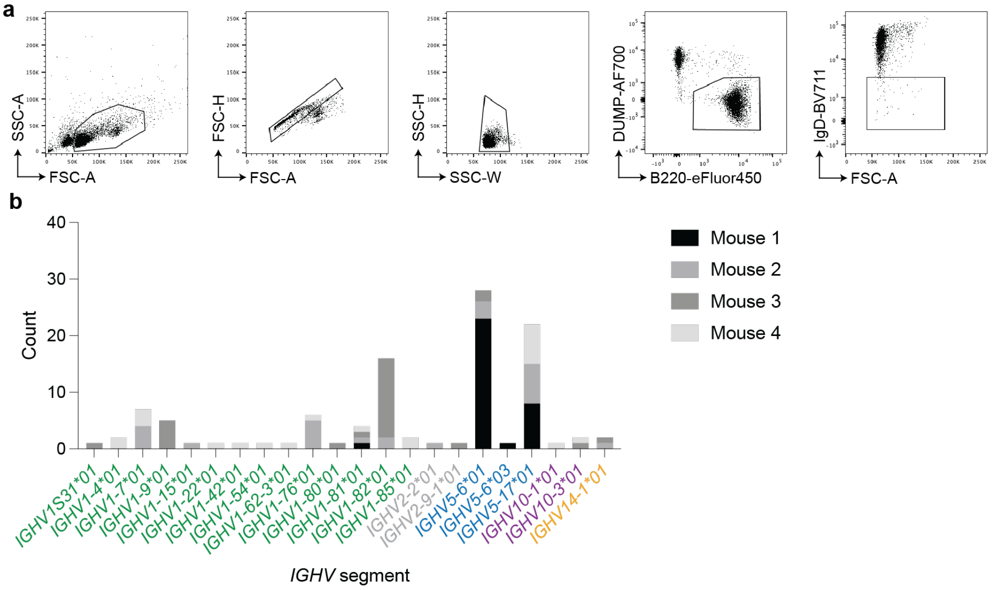

(a) Flow cytometry pre-gating strategy for mouse IgD$^-$ B cells. **(b)** V_H gene segments utilized among B cells sorted as PenG-specific. Data pooled from the four mice sequenced.

Figure S8: Analysis of CDRH3 in PenG-specific B cell clones.

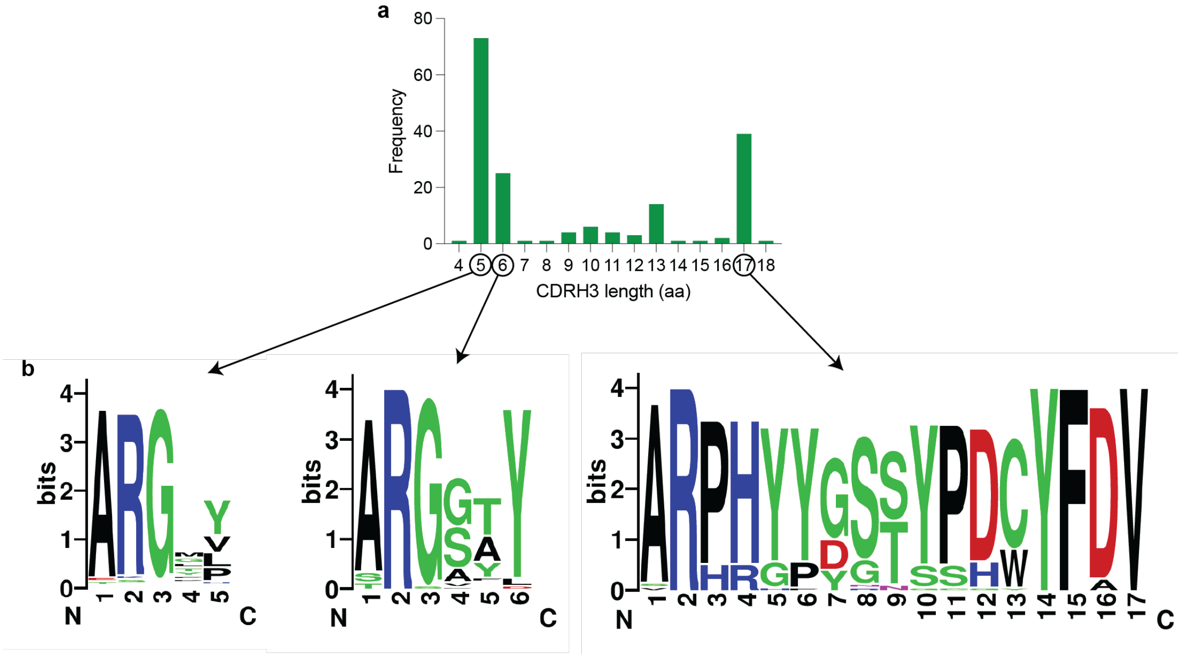

(a) Length of CDRH3s of PenG-specific B cells. **(b)** Logo plots of the CDRH3 amino acid sequence from the three most common lengths.

Figure S9: Germinal center trees of large families isolated from Mouse 1.

a

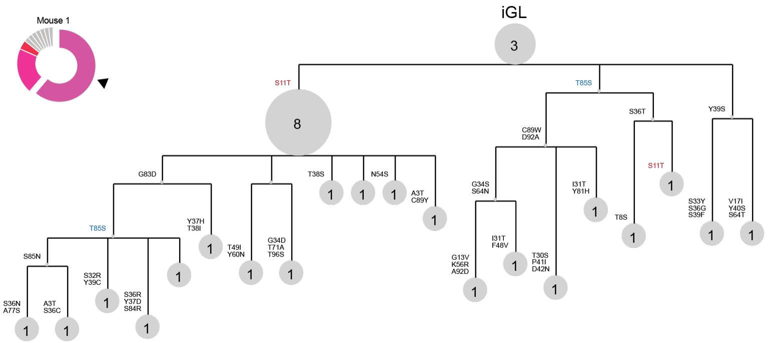

b

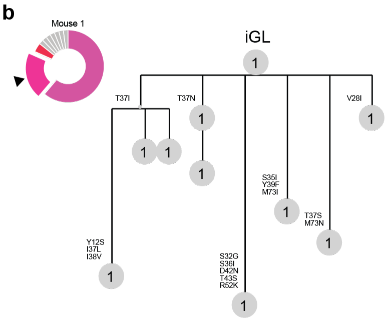

(a,b) Germinal center trees of large clonal families isolated from Mouse 1. Number and size of the nodes represents the number of B cells isolated. Amino acid residue mutations are marked.

Figure S10: MIL-3 in complex with PenG-Lys x-ray structure.

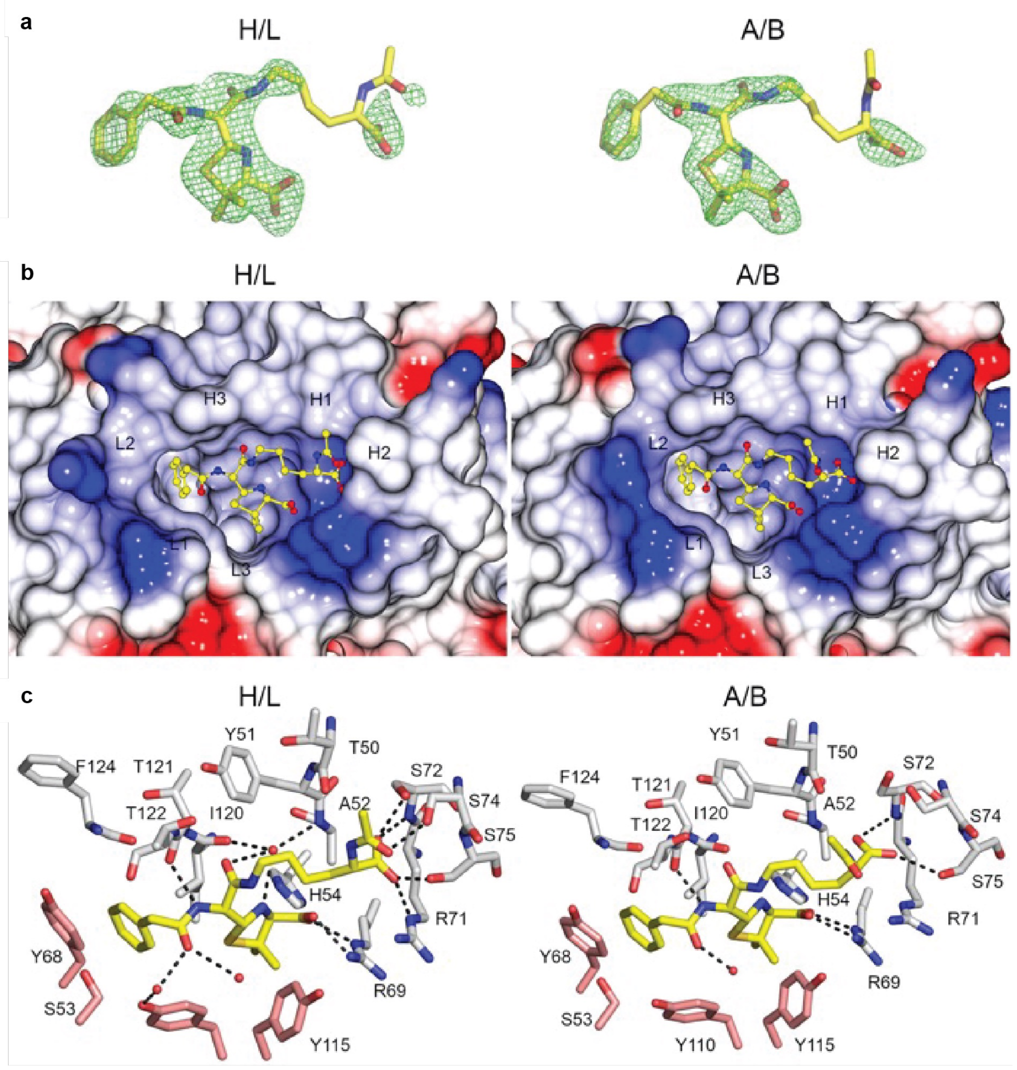

(a) F_O–F_C electron density omit map at 3 σ around PenG-Lys molecule. PenG-Lys is shown as sticks with carbon atoms coloured in yellow, nitrogen in dark blue and oxygen in red. **(b)** Surface of the binding side of MIL-3/ PenG-Lys complex structure. The surface of BMIL-3 is colored by electrostatic charges calculated in CCP4MG (red for negative potential, white for neutral and blue for positive). PenG-Lys is shown as sticks with the carbon in yellow. CDR loops have been labelled**.** **(c)** Binding site of PenG-Lys. Residues within 4.0 Å of the polysaccharide are displayed and hydrogen bonds are shown as black broken lines. PenG-Lys is shown as sticks with carbon atoms coloured in yellow. Carbon atoms of residues of chain H and L are in white and salmon, respectively.

Figure S11: MIL-3 with PenG-Lys ligplot diagrams.

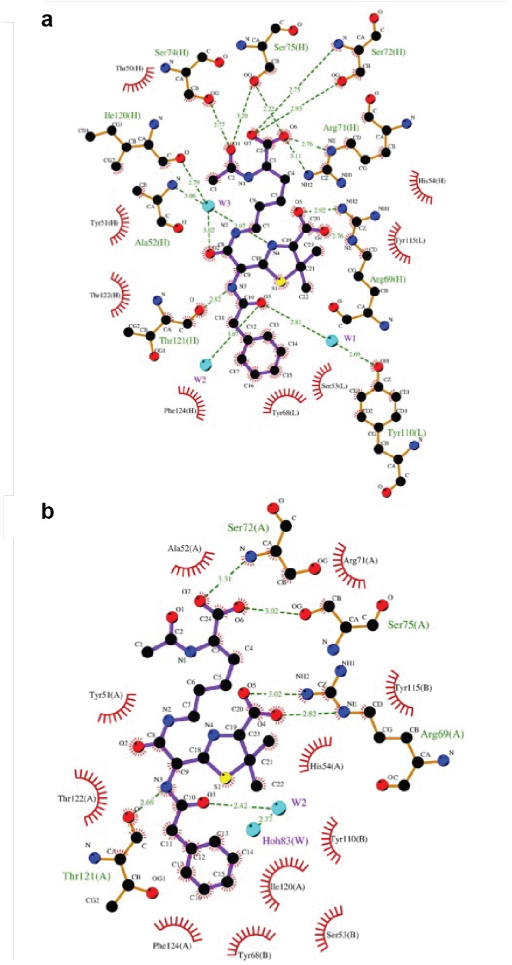

Ligplot diagrams illustrating BAR-1/Lys–C(NH)NH-GM3g interactions for chain H/L **(a)** and A/B **(b)**. Covalent bonds of the polysaccharide and the protein residues are in purple and brown sticks, respectively. Hydrogen bonds are represented by green dashed lines and hydrophobic contacts are shown as red semi-circles with radiating spokes.

Figure S12: Determining the drug-sequestering effects of the PenG-specific antibody response.

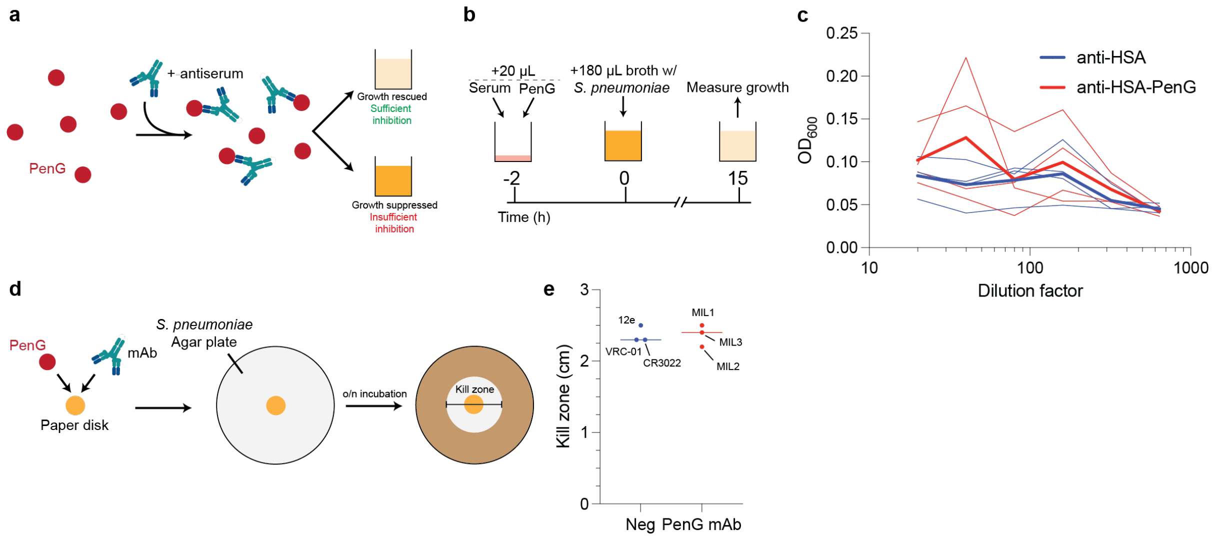

(a) Methodological concept for liquid antibiotic rescue assay. **(b)** Culture strategy. **(c)** Growth response to pre-incubated antiserum and PenG. Thin lines represent serum from a single mouse (n =4), and the thick line denotes the median. **(d,e)** Disk diffusion assay. Clone identifications, as marked. **(a,b,d)** Created with BioRender.com released under a Creative Commons Attribution-NonCommercial-NoDerivs 4.0 International license.

	8QXC
Data collection	
Space group	P 1 21 1
Cell dimensions	
a, b, c (Å)	81.57, 113.23, 82.87
α, β, γ (°)	90.0, 108.80, 90.0
Resolution (Å)	66.84-2.3 (2.34-2.3)
Rsym or Rmerge	0.149 (1.049)
I / σ(I)	8.4 (0.6)
Completeness (%)	99.8 (95.2)
Redundancy	7 (6.4)
CC half	0.991 (0.623)
Refinement	
Resolution (Å)	66.84.24-2.3
No. reflections	63174
Rwork / Rfree	0.22/0.27
No. atoms	
Protein	9736
Ligand/ion	72
Water	82
B-factors	
Protein (min-max)	49.78 (42.26 for L; 59.28 for C)
Ligand (min-max)	55.95 (47.66 for H; 64.23 for A)
Water	40.4
R.m.s deviations	
Bond lengths (Å)	0.009
Bond angles (°)	1.28

Each dataset was collected from a single crystal. *Values in parentheses are for highest-resolution shell.

Table S1: Crystallographic data and refinement statistics

Supplementary Document 1

1.Synthesis of penicillin-lysine ligand 1

General methods

All reagents were purchased from commercial sources and were used without further purification unless noted. Dry solvents for reactions were purchased from Fisher Scientific, where the solvent product series of ’[Solvent name], 99.9 % Extra Dry, AcroSeal^{TM}’ were used for all reactions. The following abbreviations are used: PE = petroleum ether (b.p. 40 – 60 °C), EtOAc = ethyl acetate, THF = tetrahydrofuran. Thin Layer Chromatography (TLC) was carried out using Merck aluminium-backed sheets coated with Kieselgel 60-F254 silica gel. Visualization of the reaction components was achieved using UV fluorescence (254 nm) and/or by charring with an acidified p-anisaldehyde solution in ethanol or an acidified cerium ammonium molybdate (CAM) solution in water. Organic solvents were evaporated under reduced pressure. Lysine-penG product was purified by a Teledyne Combiflash Nextgen 300 system with a 50 g HP C18 Gold cartridge. The gradient for Combiflash purification is: 5% B at 0 CV, 5% B at 1 CV, 50% B at 11 CV, 100% B at 14 CV, 100% B at 16 CV (Solvent A = Milli Q water, solvent B = acetonitrile). Proton Nuclear Magnetic Resonance (^{1}H NMR) spectra were recorded on a Bruker AVB400 (400 MHz), or AV700 (700 MHz) spectrometers, and the chemical shifts are referenced to residual $CHCl_3$ (7.26 ppm, $CDCl_3$), CHD_2OD (3.30 ppm, CD_3OD), HDO (4.79 ppm, D_2O). Carbon Nuclear Magnetic Resonance (13C NMR) spectra were recorded on a Bruker AVB400 (100 MHz), or AV700 (175 MHz) spectrometers and are proton decoupled, and the chemical shifts are referenced to $CDCl_3$ (77.16 ppm) or CD_3OD (49.0 ppm). Assignments of NMR spectra were based on two-dimensional experiments (^{1}H-^{1}H COSY, DEPT-135, HSQC, and HMBC) if required. Reported splitting patterns are abbreviated as s = singlet, d = doublet, t = triplet, q = quartet, p = pentet, hept = heptet, m = multiplet, br = broad, app = apparent. Low Resolution Mass Spectra (LRMS) were recorded on a Waters ACQUITY QDA mass spectrometer using electrospray ionization (ESI). High Resolution Mass Spectra (HRMS) were recorded on a Waters Xevo-G2 QTof spectrometer using electrospray ionization (ESI), m/z values are reported in Daltons.

Synthesis of (2R,4S)-2-((R)-2-(((S)-5-acetamido-5-carboxypentyl)amino)-2-oxo-1-(2-phenylacetamido)ethyl)-5,5-dimethylthiazolidine-4-carboxylic acid (**1**)

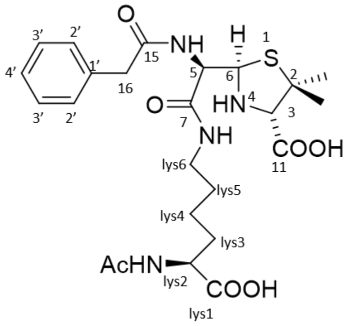

1

Penicillin G sodium salt (20 mg, 0.056 mM) and Nα-*acetyl*-L-*lysine (30 mg, 0.159 mM)* were dissolved in 0.5 mL sodium carbonate buffer (0.1 M, pH = 11). The solution was stirred at room temperature overnight. When TLC showed the completion of reaction, the entire reaction mixture solution was injected into a C18 combiflash reverse phase purification system (see General Methods for more details). Fractions containing the product was combined and passed through a small column of Amberlite IR-120 resin (Na^+ form). The solvent was removed under vacuum to afford the product as a white solid (22 mg, 75%). Chemical evaluation shown in **Fig S13**, **Fig S14** and **Fig S15**.

Characterisation: TLC: R_f = 0.5 (2:2:1 EtOAc: isopropyl alcohol: water). ^{1}H NMR (400 MHz, D_2O) δ 7.32 (dd, *J* = 8.0, 6.4 Hz, 2H, H3'), 7.31 – 7.20 (m, 3H, H2' & H4'), 4.82 (d, *J* = 9.3 Hz, 1H, H6), 4.20 (d, *J* = 9.3 Hz, 1H, H5), 3.99 (dd, *J* = 9.2, 4.5 Hz, 1H, Lys-H2), 3.64 – 3.51 (m, 2H, H16), 3.40 (s, 1H, H3), 3.15 – 2.99 (m, 2H, Lys-H6), 1.94 (s, 3H, Lys-AcNH), 1.68 (dtd, *J* = 13.2, 7.7, 4.6 Hz, 1H, Lys-H3), 1.51 (s, 3H Me2 + Lys-H5a), 1.37 (dtt, *J* = 62.6, 13.4, 6.6 Hz, 1H, Lys-H5), 1.23 (q, *J* = 7.9 Hz, 2H, Lys-H4), 1.17 (s, 3H, Me1).^{13}C NMR (101 MHz, D_2O) δ 179.51, 174.97, 174.44, 173.54, 171.10, 160.89, 134.89, 129.13, 128.91, 127.31, 74.37, 64.62, 59.35, 59.25, 55.27, 42.02, 39.04, 31.16, 27.90, 27.15, 26.73, 22.71, 22.00. HRMS (ESI/Q-TOF): *m/z* [M+H$^+$] Calculated: 523.2226 Found: 523.2247.

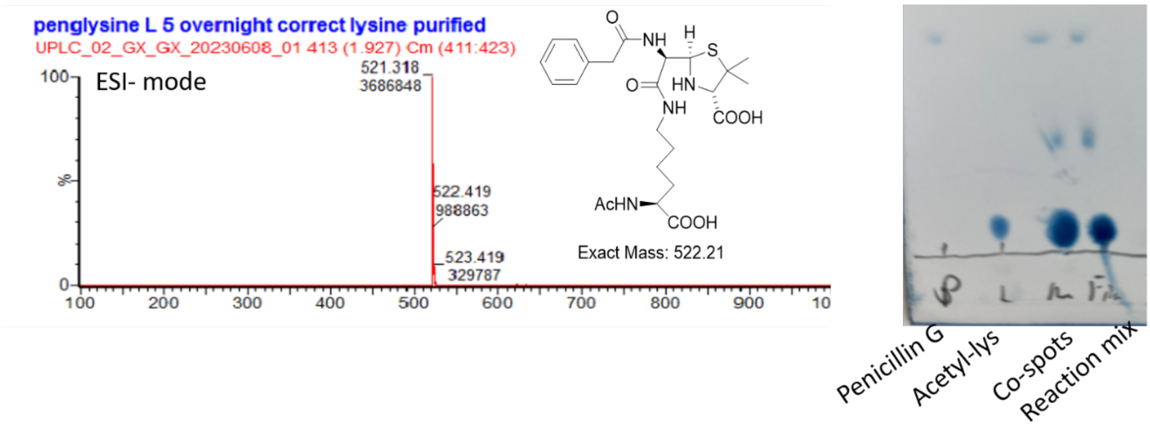

Figure S13: Mass spectral and TLC chromatographic analysis of PenG-Lys. Low resolution MS (left) and example TLC for the reaction (right, 2 h time point).

^{1}H NM

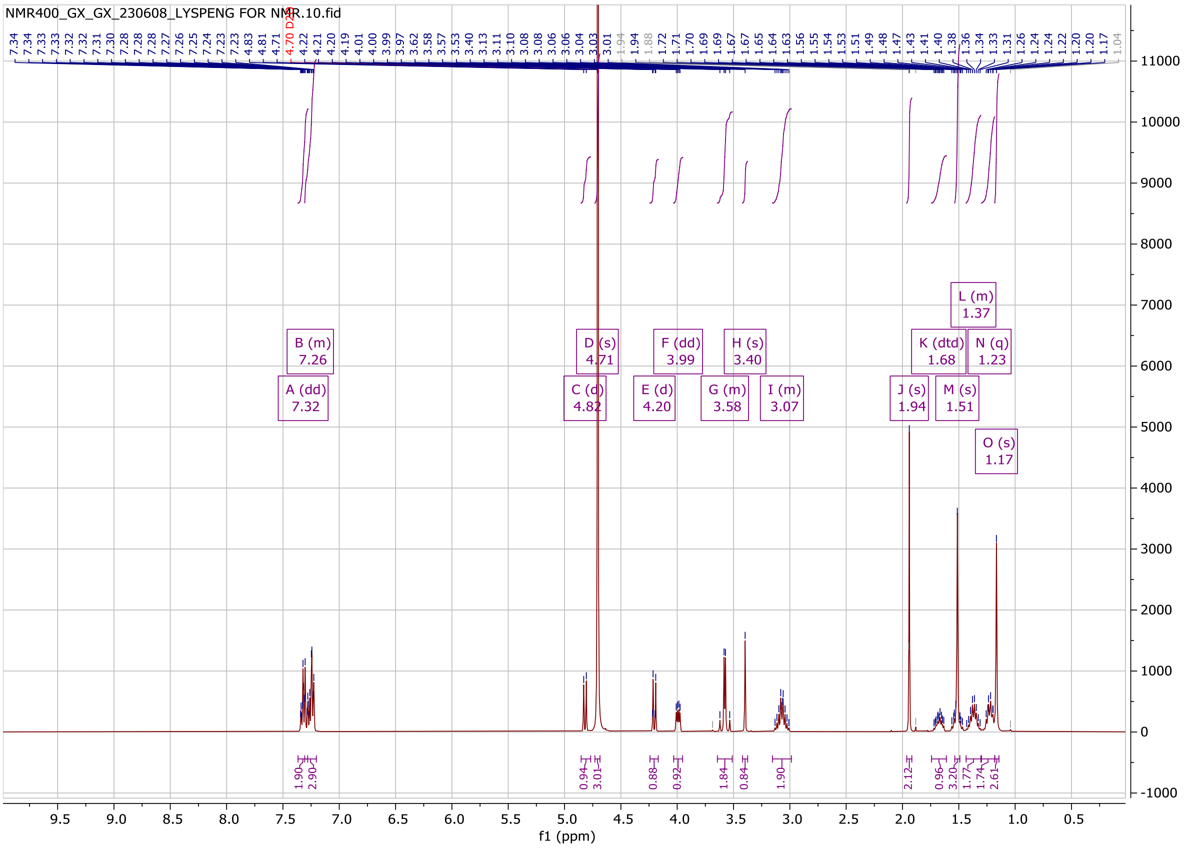

^{13}C NMR

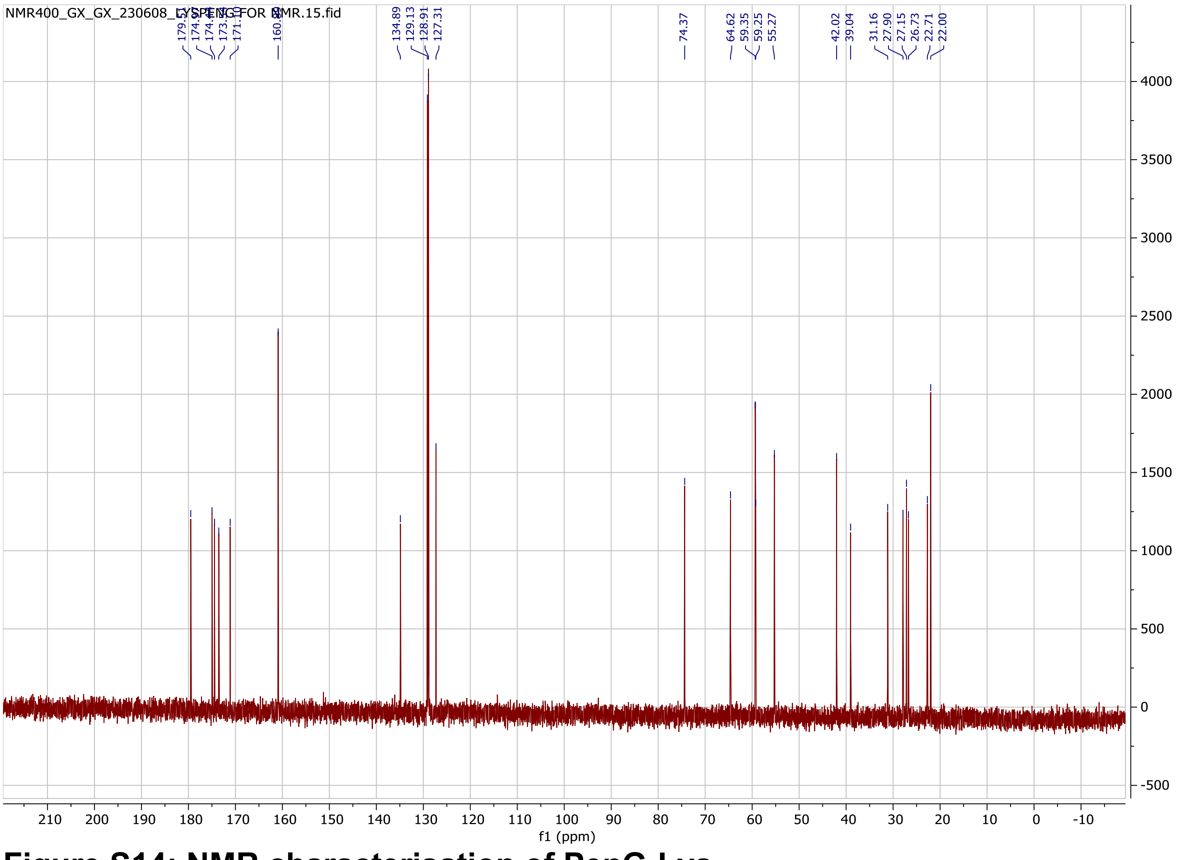

Figure S14: NMR characterisation of PenG-Lys.

^{1}H (top) and ^{13}C (bottom) NMR characterisation of **1**.

HMBC NMR

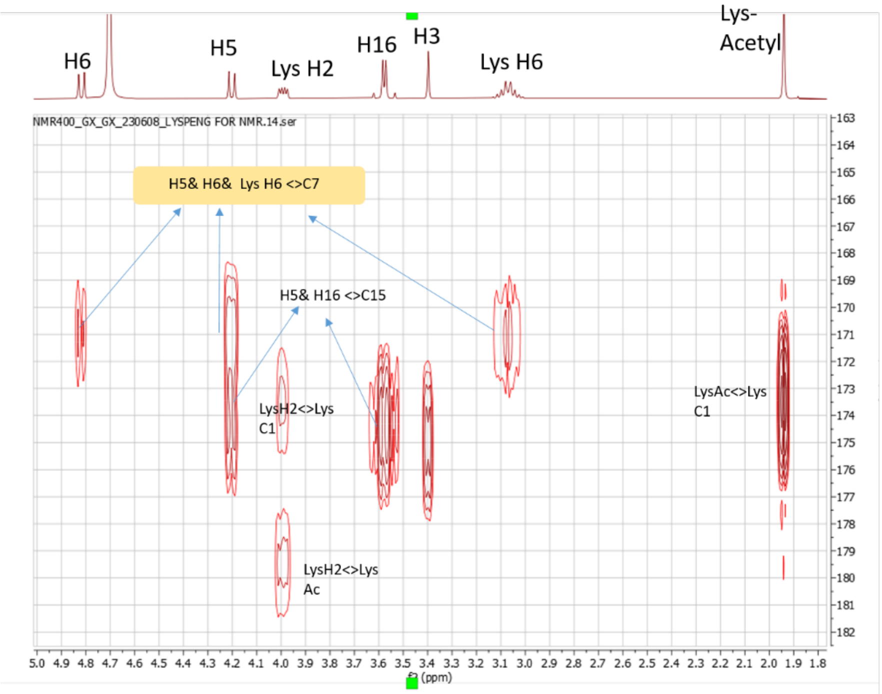

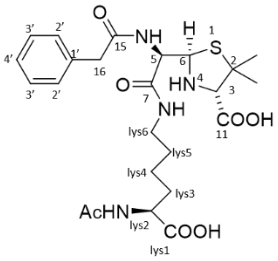

HRMS

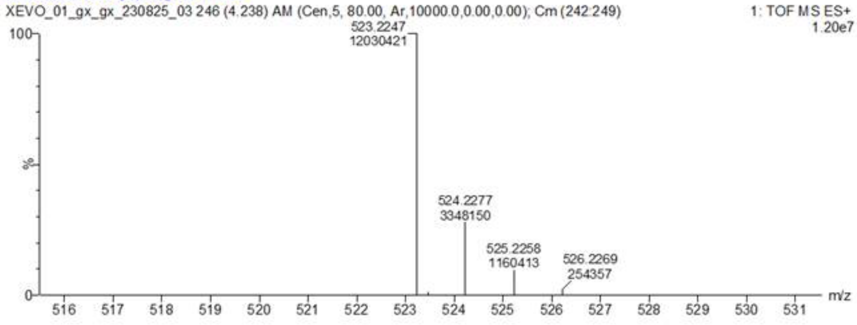

Figure S15: HMBC and HRMS analysis of PenG-Lys.
(top) HMBC NMR analysis showing the ring-opening and ε-amide linkage between lysine and penicillin G; LysH6 is coupled with C7 of penicillin G. (bottom) HRMS of PenG-Lys.

2. Preparation of PenG-lysine SPR chip

PenG-lysine stability test

We carried out a small-scale stability test to find the best storage condition for the penG-lysine SPR chip. Experimental design: 3 mg of compound **1** was dissolved in 700 uL D_2O PBS and the pH was adjusted to 2, 8 and 11. These 3 samples were analysed over time by ^1H NMR (400 MHz). Data shown in **Fig S16**

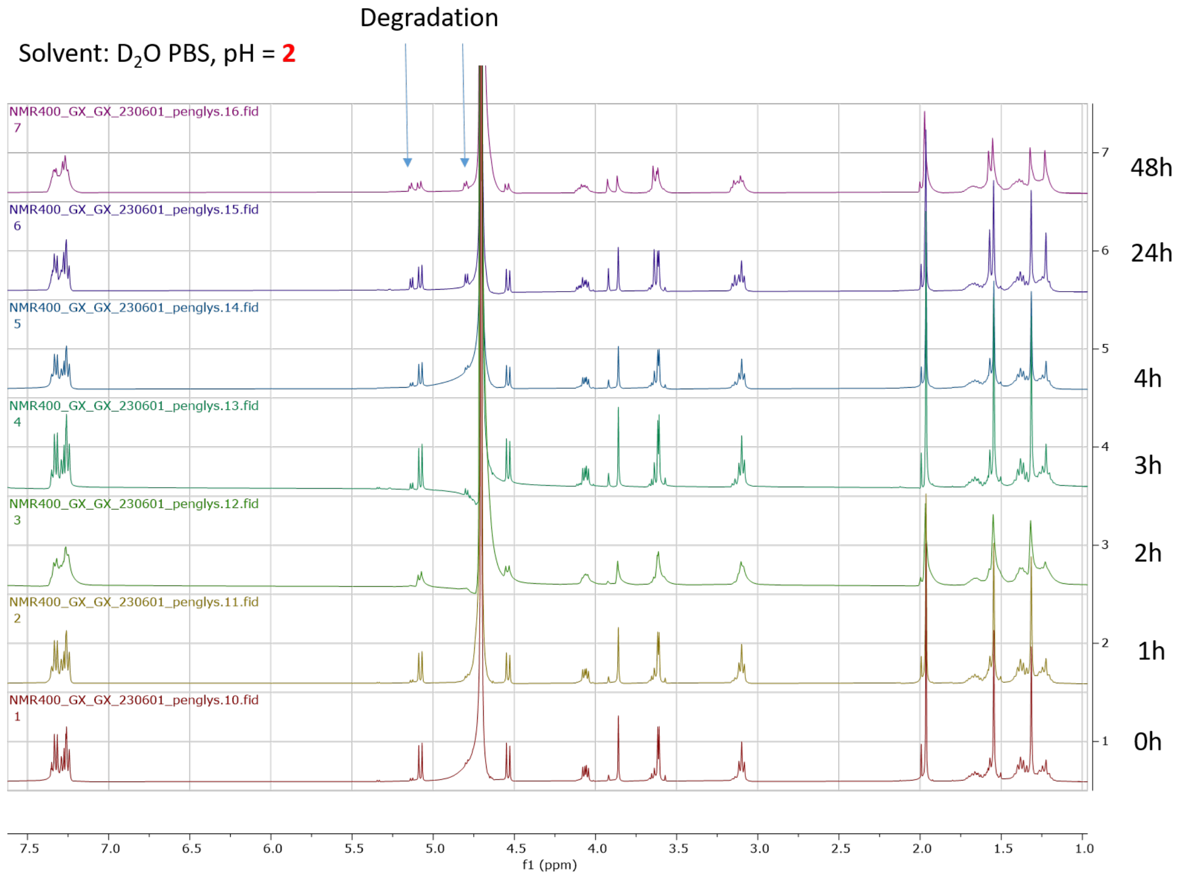

Figure S16: Stability test of PenG-lysine (1).
Samples were prepared in D_2O PBS and pH was adjusted to relevant value showed in the figure. We only observed degradation at pH = 2. Compound 1 in pH = 11 & 7.5 D_2O PBS is stable and guided the storage of penicillin G - adduct SPR chip.

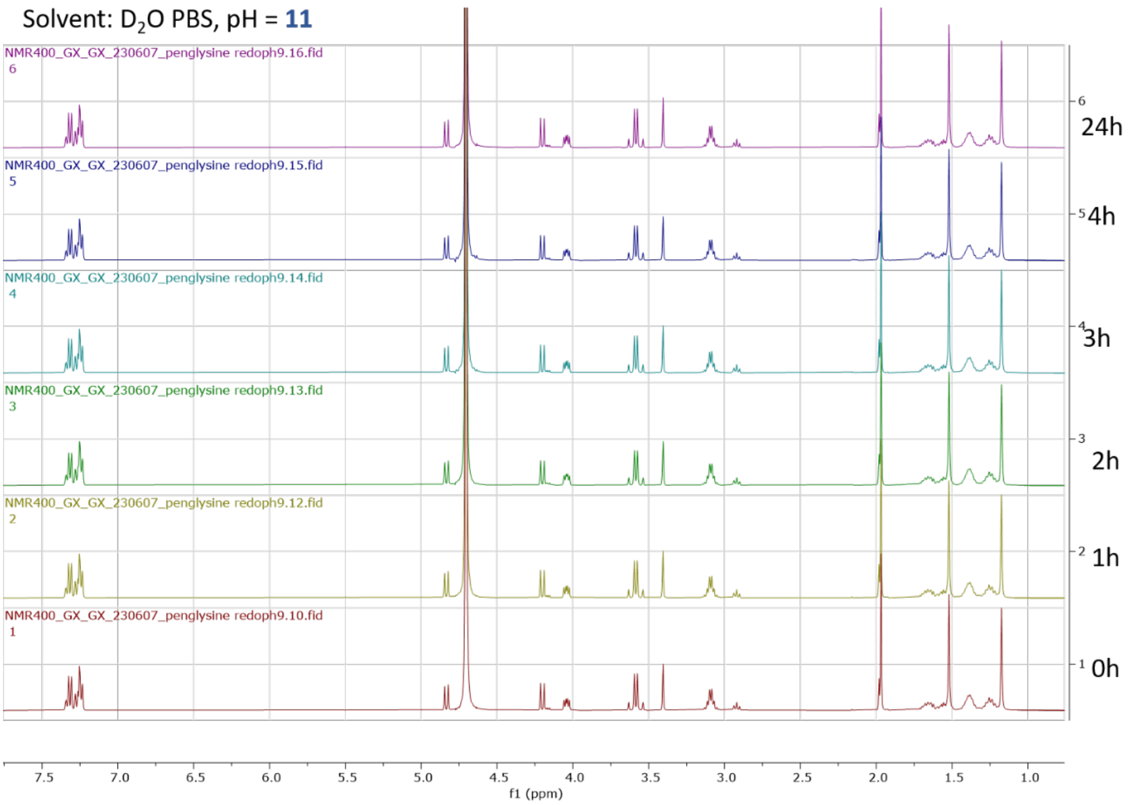

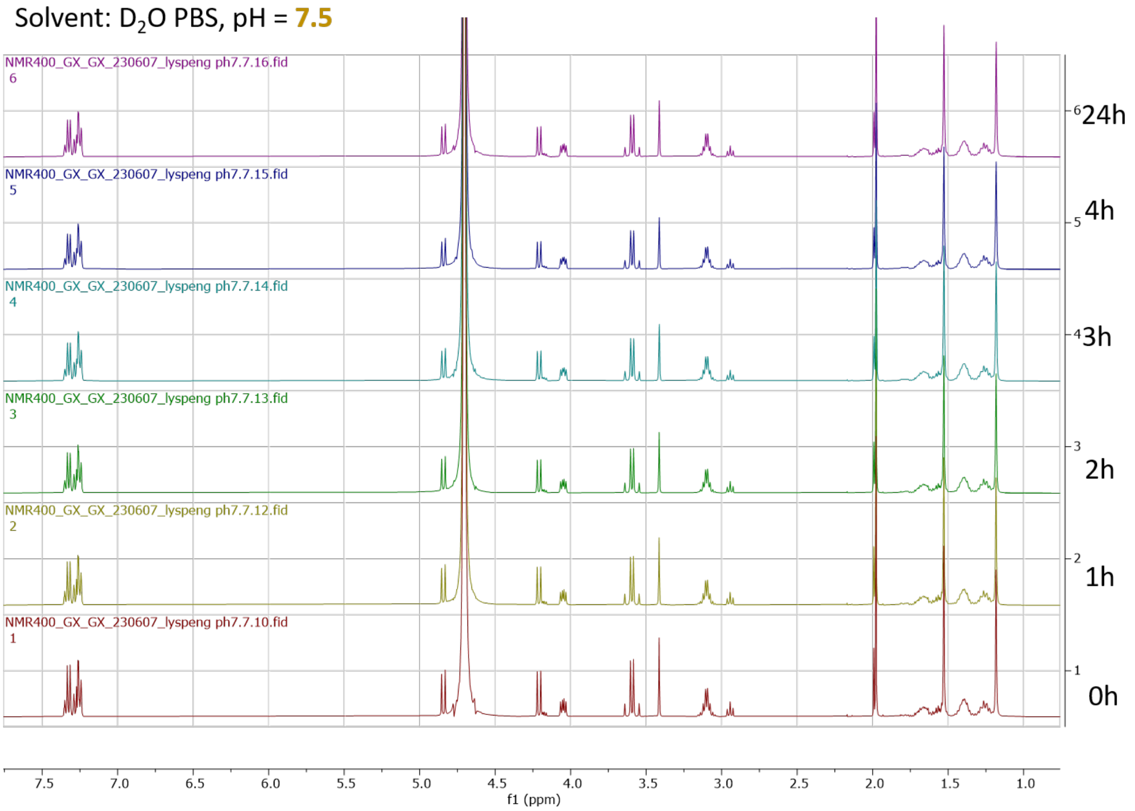

Figure S16: Stability test of PenG-lysine (1). (Cont.)

Preparation of penicillin G SPR chip

Immobilisation of penicillin-G onto carboxymethylated SPR sensor chip was conducted (**Fig S17**). **A** carboxymethylated CM5 sensor chip was activated with *N*-hydroxy-succinimide (NHS) and *N*-ethyl-N′-(3-dimethylaminopropyl)carbodiimide hydrochloride (EDC•HCl) and subsequently coupled to ethylenediamine followed by blocking of unreacted esters with ethanolamine. Penicillin G was dissolved in sodium carbonate buffer (pH = 11) and was then injected into the flow cell for in situ reaction with surface-bound free amines, mimicking the amide adducts penicillin G-lysine.

Specifically, in step i, a CM5 chip was activated with NHS (50 mM) and EDC (200 mM) for 10 min at 10 μL/min. In step ii, ethylenediamine (1 M) was injected to functionalise chip with free amine groups for 7 min at 10 μL/min. Then ethanolamine (1 M) was injected over 10 min to block any unreacted NHS activated esters at 10 μL/min. In step iii, penicillin G sodium (112 mM) was injected over 150 min at 5 μL/mL followed by washing with PBS buffer (wash steps with buffer between each step and during sensor equilibration). The prepared chip was stored in PBS (pH = 8) to prevent dry out and degradation.

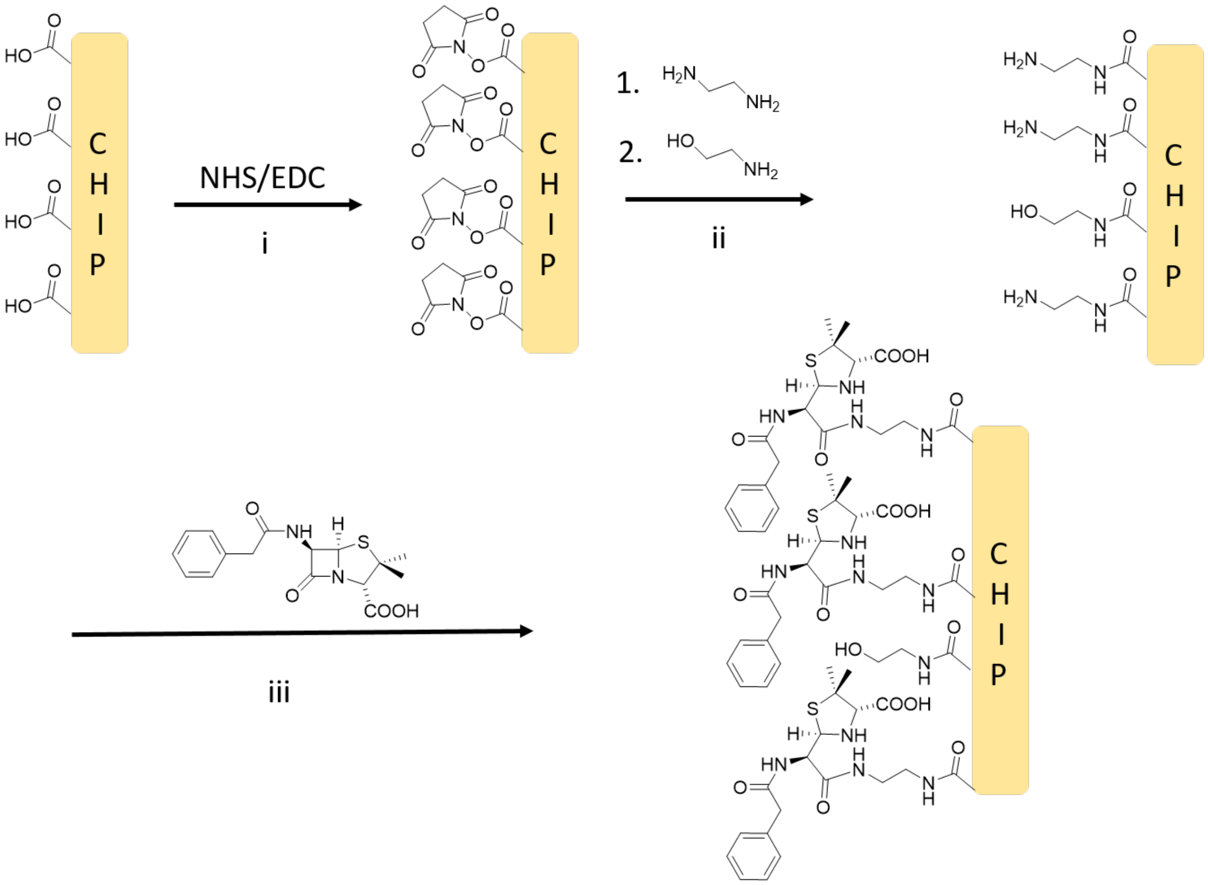

Figure S17: Preparation of penicillin G SPR chip.
Stepwise chemical reaction pathway for the generation of the PenG SPR chip.

3. Preparation of protein-PenG antigen.

Protein adduct formation was adapted from [1] where reaction was conducted in 0.1 M Na_2CO_3 solution. We selected hen egg lysozyme (HEL) as a model protein for this reaction. We also prepared samples at different pHs to define the optimum pH.

Experimental

0.1 M Na_2CO_3 solutions ranging across pH (11, 10, 9, 8, 7.8, 7.6, 7.4, 7.2, 7.0, 6.8) were prepared. For each was added: 0.2 mg HEL commercial HEL protein powder and then penicillin G sodium salt (200 eq. per-lysine) at a final concentration of protein of 1 mg/mL. The solution was stirred overnight at room temperature and modification assessed by intact protein LC-MS (unmodified HEL = 14306 Da).

Results:

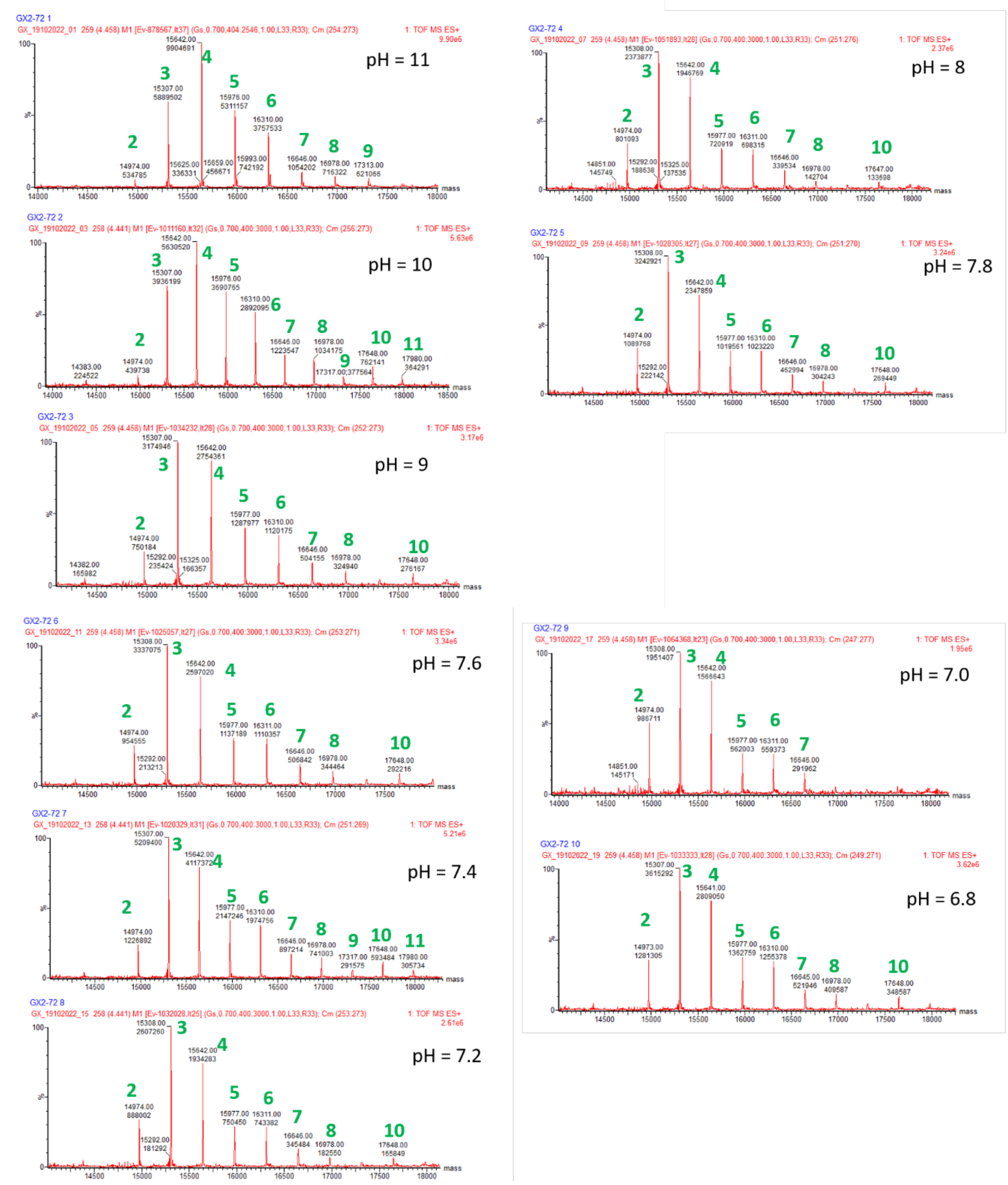

Figure S18: Penicillin G modified HEL at different pHs in carbonate solution. Copy number of penicillin molecule loaded to the HEL protein shown in green, i.e.. 2 modification means: 14306 Da (Mass of naked HEL protein) + 334 Da (Mass of penicillin G) x 2 = 14974 Da.

Discussion and Conclusion (Fig S18): Sodium carbonate solution was preferred for penicillin G-protein complex preparation, as it resulted in at least 2 modifications and up to 11. Change of pH did not greatly affect the modification extent under these conditions. It should be noted that the generation of > 7 adducts suggests potential reactivity of other, non-lysine type protein residues also. All reactions described in the paper were conducted in carbonate solution at pH = 8.

Additional evaluation of non-carbonated buffers:
We repeated the penicillin-HEL reaction described in previous section but also tested the use of Milli Q water alone (**Fig S19a**) and commercial PBS buffer (pH = 7.2, **FigS19b**) to investigate the effect of carbonate.

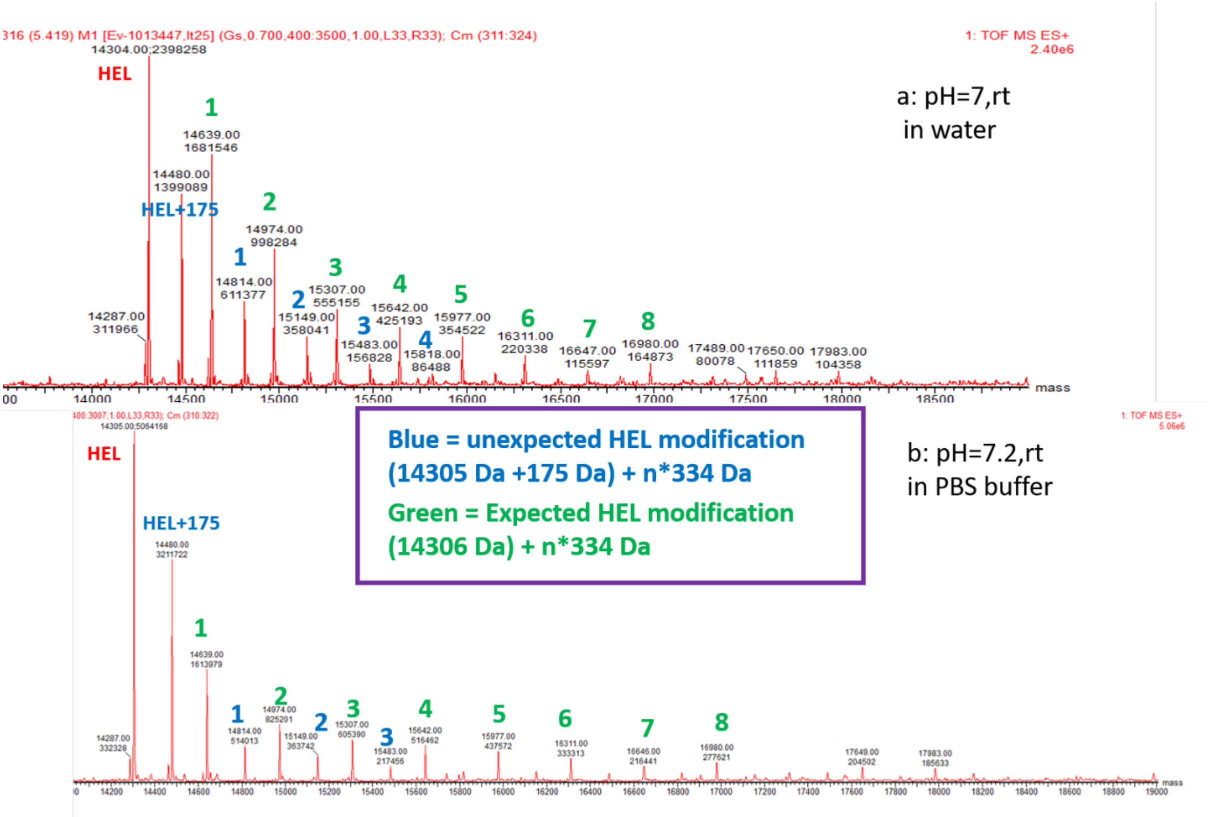

Figure S19: Penicillin G-modified HEL in Milli Q water and PBS.
Modification of HEL conducted in (a) H2O (pH = 7) or (b) PBS (pH = 7.1). Green numbers represent the number of penicillin molecules adducted to HEL protein, i.e.. 2 represents 14306 Da (Mass of naked HEL protein) + 334 Da (Mass of penicillin G) * 2 = 14874 Da. Blue numbers represent unexpected HEL modification from the initial HEL + 175 Da modification.

The reaction proceeded more slowly in water and PBS, with a significant amount of unreacted HEL protein remaining after an overnight incubation (Figure 3, peak 14305 Da). Surprisingly, we also observed a group of patterned and unexpected peaks, where the naked HEL protein was firstly modified by an unknown adduct mass of 175 Da (= HEL 14035 Da + 175 Da), as well as an associated adduct series arising from additional PenG reaction. As a result, the synthesis of penicillin-G antigen was restricted to carbonate solution at pH = 8.

4. Additional References

[1] Padovan, E., Bauer, T., Tongio, M.M., Kalbacher, H. and Weltzien, H.U., 1997. Penicilloyl peptides are recognized as T cell antigenic determinants in penicillin allergy. *European Journal of Immunology*, *27*(6), pp.1303-1307
